# Interrelationships between Multiple Climatic Factors and Incidence of Foodborne Diseases

**DOI:** 10.3390/ijerph15112482

**Published:** 2018-11-07

**Authors:** Myoung Su Park, Ki Hwan Park, Gyung Jin Bahk

**Affiliations:** 1 Department of Food and Nutrition, Kunsan National University, Gunsan 54150, Jeonbuk, Korea; ijea1418@nate.com; 2 Department of Food Science and Technology, Chung-Ang University, Ansung 17546, Gyeonggi, Korea

**Keywords:** foodborne disease, climatic factors, interrelationship, network, South Korea

## Abstract

Climatic factors can affect the incidence of foodborne diseases (FBDs). Moreover, microbial network inference is useful for predicting the interrelationships between the incidence of FBDs and climatic factors. However, the interrelationships between FBD pathogens and most climatic factors are unknown. Using principal component analysis (PCA) and partial correlation coefficient matrices (PCCMs), we determined the intra-ecosystem interrelationship network of the multiple combined effects of 5 climatic factors (temperature, relative humidity, rainfall, insolation, and cloudiness) and the monthly incidences of 12 bacterial FBDs. Many FBD pathogens are interrelated with multiple combined factors. Salmonellosis has strong positive interrelationships with *Vibrio parahaemolyticus* and enterohemorrhagic *Escherichia coli*, and the interrelationships between *Staphylococcus aureus*/enteropathogenic *E. coli*/enterotoxigenic *E. coli* exhibits a typical triangular pattern with the combined effects of all 5 climatic factors. Meanwhile, campylobacteriosis and *Clostridium perfringens* infections are negatively interrelated with insolation and cloudiness. Enteroinvasive *E. coli*, *Bacillus cereus*, *Listeria* spp., and *Yersinia enterocolitica* are significantly interrelated with any climatic factor combination. The interrelationships or higher-order interrelationships among these climatic factors play an important role in the incidence of FBDs, although the underlying mechanisms remain unclear. Our results will serve as a foundation for more sophisticated models of future FBD patterns with regard to climate change.

## 1. Introduction

Climatic factors such as ambient temperature, rainfall, and humidity can affect the incidence of several foodborne diseases (FBDs) [1,2]. FBD incidences are therefore likely to be affected by climate change [3]. However, most studies focused on specific climatic factors for specific bacterial FBD pathogens [4,5,6,7,8,9,10,11]. In nature, these bacterial FBD pathogens and climatic factors do not exist alone, but rather form complex ecological interrelationship webs. Therefore, there may be many distinct ecological interrelationships among these FBD-causing microbial pathogens and related climatic factors [2]. In addition, examining the associations between regional climate linked to FBDs will develop our understanding of climatic triggers for enteric infections as well as improve disease forecasts [12].

While locally specific effects of climate change on disease risk will depend on a number of interacting climatic and non-climatic factors, larger-scale, regional disease patterns are more likely to be dominated by extrinsic climate factors [13]. Such climatic variations are likely to affect FBD incidence either directly, through effects on pathogen competence, or indirectly, by influencing transmission pathways and host behavior [14,15]. In particular, interrelationships between FBDs and foodborne pathogens are striking features of the climate system which appear as statistically correlated climate-related patterns between geographical regions [16]. However, the interrelationships between FBD cases caused by foodborne pathogens and the majority of climatic factors remain poorly understood.

Recently, regarding ecological interrelationships in microbial communities, co-occurrence and correlation patterns are increasingly being used to predict species interrelationships in environments ranging from the nature to the human microbiome [17]. However, to date, there are no published studies taking a comparative approach to assessing the interrelationship of such large-scale multiple climatic factors across FBDs over an entire region. Especially, understanding the interrelationship network of the combined influence of climate variability on FBDs may improve our ability to predict the effects of climate change on these diseases [11]. Therefore, this study could allow better evaluation of the effects of climate variability and change on FBD risk, and an assessment of the effect of climate factors on human health is very important to establish national environmental health policies.

The aim of this study is to predict the ecological interrelationships among FBD pathogens and climatic factors on the basis of the incidence of FBDs; hence, we investigated intra-ecosystem interrelationships including the multiple combined effects of 5 climatic factors (temperature, relative humidity, rainfall, insolation, and cloudiness) and the monthly incidence (hospitalization) of 12 bacterial FBD pathogens in South Korea from 2011 to 2015 using principal component analysis (PCA) and partial correlation coefficient matrix (PCCM) technique.

## 2. Methods

### 2.1. Climate Data

The 5 climatic factors analyzed were temperature (mean minimum), relative humidity, rainfall, insolation, and cloudiness. Ten climatic factors (mean minimum, mean, and mean maximum temperature; relative humidity; rainfall; snowfall; wind speed; sunshine duration; insolation; and cloudiness) were considered in the preliminary analysis, but only the 5 factors listed above were strongly correlated with FBD incidences. The data that applies to all of South Korea, as a region, were published by the Korea Meteorological Administration [18] as described by Kim et al. [19]. Because the bacterial FBD incidence data were in a monthly format, we calculated the mean monthly values of the climatic factors from January 2011 to December 2015.

### 2.2. Bacterial FBD Incidence Data

Data of bacterial FBD cases from 2011 to 2015 were obtained from the Health Insurance Review and Assessment Service (HIRA) [20] using the method of Park et al. [21], which considers diagnostic accuracy and diagnoses matched with the International Classification of Diseases, 10th Revision (ICD-10) codes for the following 12 foodborne pathogens: salmonellosis (SAL); enteropathogenic *E. coli* (EPEC), enterotoxigenic *E. coli* (ETEC), enteroinvasive *E. coli* (EIEC), and enterohemorrhagic *E. coli* (EHEC); *Vibrio parahaemolyticus* (VBR); *Staphylococcus aureus* (STA); campylobacteriosis (CAM); *Clostridium perfringens* (CLP); *Bacillus cereus* (BAC); *Listeria* spp. (LIS); and *Yersinia enterocolitica* (YER) infection. These data are also applicable locally to all of South Korea. The estimated numbers of bacterial FBD cases were subsequently grouped according to inpatient stays (i.e., hospitalizations) and outpatient visits by month. However, the pre-analyses showed stronger correlations between climatic variables and inpatient data than with outpatient data, or total outpatient and inpatient data; therefore, we used only inpatient data for the analysis, as in a previous study [3].

### 2.3. Microbial Interrelationship Network

PCA was used to reduce the effect of higher-order multicollinearity, investigate the combined effects of the above mentioned 5 climatic variables in South Korea, and investigate the structure that identifies the similarities and differences within the climatic data. As a result, the manifest variables and components set were reduced to subcomponents termed PC1, PC2, PC3, etc., which are independent and decrease the amount of variance from the original dataset: PC1 captures most of the variance, PC2 captures the next highest variance, and so on until all of the variance is accounted for [22].

Then, the PCCM technique was used to identify direct relationships between bacterial FBD cases and climatic variables. The PCCM technique has been successfully applied for various network inference problems, making it appropriate for this study [23]. Partial correlation coefficients can distinguish between direct and indirect correlations when used with suitable cutoffs. To determine the unique variance between 2 variables while eliminating the variance from other variables, the partial correlation coefficients between bacterial FBD pathogens and non-eliminated climatic variables, while controlling for PC1, PC2, or PC1+PC2 as a third variable, were calculated (Table 1).

### 2.4. Calculation of Correlation Thresholds and Statistical Analysis

To test the significance of partial correlations (i.e., cutoff calculations), the correlation coefficients were transformed into *Z*-terms. For a selected significance level (*p* < 0.01 or *p* < 0.05), the corresponding *Z*-score threshold (*Zα*/2, i.e., cutoff) was calculated [24,25]. In order to test the significance of the associations among variables, the correlation coefficients were transformed to *Z*-terms using Equation (1).

(1) $$\;Z_{ij}=0.5*\ln\left(\frac{1+r_{ij}}{1-r_{ij}}\right),\;$$

where *r_ij_* is the magnitude of a partial correlation coefficient between 2 variables: *i* and *j* (Table 1). These transformed values were subsequently used to calculate the *Z*-score as in Equation (2), which were then used for Z-statistics hypothesis testing.

(2) $$Z=\frac{Z_{ij}}{\sqrt{1/n-3-p}},$$

where *n* is the number of observations used to evaluate the correlations (*n* = 60 (5 years × 12 months) in this study) and *p* is the conditioning order of the correlation coefficient (*n* = 1 (first-order conditioning) in this study). We used 2 different *α* levels, 0.05 and 0.01, which have corresponding threshold values of *Zα*/2 = 1.96 and 2.575, respectively. The corresponding values for the minimal absolute correlation coefficients were used as cutoffs in the network determination (Table 2). All analyses were performed using SPSS version 12.0 (Data Solution Inc., Seoul, Korea). The level of significance was set at *p* < 0.05.

## 3. Results

### 3.1. PCA and PCCM

**Figure 1:**
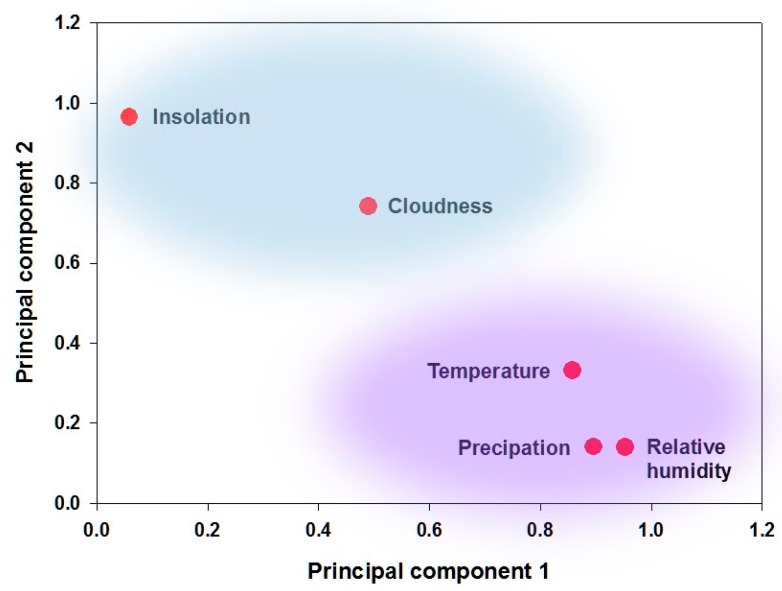
Boxplot of principal component analysis (PCA) of 5 climatic variables in South Korea from January 2011 to December 2015. PC1 (temperature, relative humidity, and rainfall) and PC2 (insolation and cloudiness) accounted for 65.2% and 21.2% of the total variance, respectively.

PCA can be used to identify patterns, highlighting similarities and differences in a dataset, and to explore the structure of weather data in South Korea. The present PCA can be divided into 2 new independent components termed PC1 (temperature, relative humidity, and rainfall) and PC2 (insolation and cloudiness) to decrease the variance of the original dataset; these components are considered single climatic factors. PC1 captured most of the variance (65.2%), followed by PC2 (21.2%) (Figure 1). Consequently, the interrelationship network of bacterial FBD pathogens with respect to multiple climatic factors was created with 3 distinct conditional climatic variables: PC1 + PC2, PC1, and PC2.

The PCCM showing the interrelationship among 12 bacterial FBD pathogens and 3 conditional climatic variables (i.e., PC1 + PC2, PC1, and PC2) with α levels of 0.01 and 0.05 is shown in Table 2. The absolute values of the partial correlation coefficients ranged from 0.0015 to 4.54. Of 198 coefficients, only 25 (12.6%) were statistically significant from zero order; thus, 173 edges were removed from the networking graph.

### 3.2. Interrelationships between FBD Pathogens and Climatic Factors

**Figure 2:**
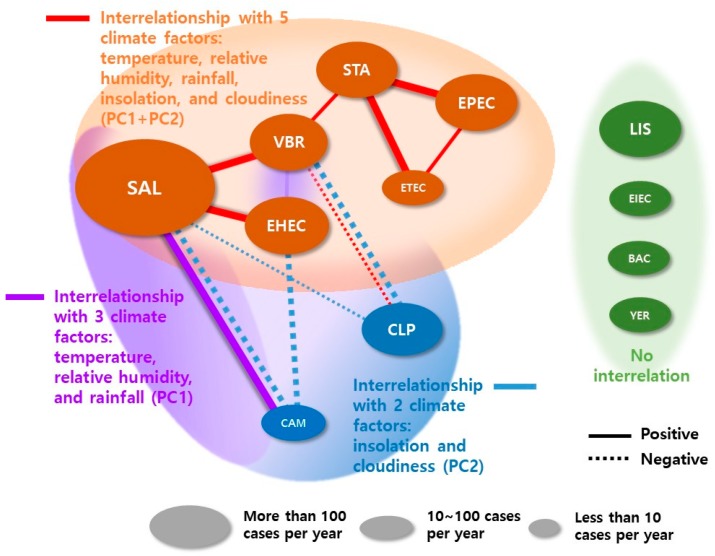
Microbial interrelationship network inferred from the monthly incidence of 12 bacterial FBDs according to climatic variables. Nodes represent the incidence of a bacterial FBD; the size indicates the number of cases per year. Lines represent significant pairwise associations between pathogens; thick and thin lines: *p* < 0.01 and <0.05, respectively. Red, purple, and blue lines show the interrelationship under the 3 conditional climatic variables: PC1 + PC2, PC1, and PC2, respectively. BAC: *B. cereus*; CAM: campylobacteriosis; CLP: *C. perfringens*; LIS: *Listeria* spp.; SAL: salmonellosis; EPEC, ETEC, EIEC, EHEC: pathogenic infection by enteropathogenic, enterotoxigenic, enteroinvasive, enterohemorrhagic *E. coli*, respectively; STA: *S. aureus*; VBR: *V. parahaemolyticus*; YER: *Y. enterocolitica* infection.

The main interrelationships of bacterial FBD pathogens with multiple climatic factors are shown in Figure 2. The SAL and VBR nodes had the most edges with 5; followed by the EHEC, STA, CAM, and CLP nodes with 3; and the EPEC and ETEC nodes with 2.

With the most patients (i.e., largest circle (i.e., node) in Figure 2), SAL strongly positively interacted with VBR and EHEC, and CAM under the PC1 + PC2 and PC1 conditions, respectively; meanwhile, it negatively interacted with CAM and CLP only under the PC2 condition. Under the PC1 + PC2 condition, STA-EPEC-ETEC interrelationships exhibited a typical triangular pattern, STA-VBR interacted positively, and VBR-CLP interacted negatively. EHEC interacted positively with VBR only under PC1 and strongly negatively interacted with CAM under PC2. VBR-CLP also interacted strongly negatively under PC2. Meanwhile, EIEC, BAC, LIS, and YER did not significantly interact with any combination of climatic factors.

## 4. Discussion

### 4.1. Application of PCA and PCCM

The present study used PCA and PCCM technique to demonstrate the interrelationship network among FBD pathogens and climatic factors, in which one pathogen depends on multiple other pathogens. The PCA results show that some climatic variables in South Korea are closely associated; thus, in order to avoid too many correlations, the 5 variables were divided into 2 components: PC1 and PC2. PCA reduces and extracts the dimensionality of data and rates the variation present in the original dataset as much as possible [23]. The results of PCA in the present study indicate a combined effect between climatic variables and the incidences of 12 bacterial FBDs in South Korea.

Furthermore, for the first time, this study applied the PCCM technique to a climate ecosystem as a preliminary approach to represent bacterial FBD pathogen interrelationships. This approach has been successfully developed and applied to interrelationship network [26]. Partial correlation networks are represented by partial correlation coefficients calculated for pairs of variables when all other variables are taken into account. In addition, partial correlation analyses may enable the identification of the direction of a partial correlation, which enables us to distinguish between the response variables and covariates [27]. Faust and Raes (2012) [17] state that one of the pitfalls of network construction is the faulty prediction of a relationship between 2 species, because both are inevitably affected by a third one. To overcome this issue, tests for conditional independence (e.g., partial correlation) are commonly applied to exclude these indirect links [28,29]. This more complex approach, i.e., not determining simple pairwise relationships, enabled the identification of the positive and negative interrelationships between the climatic factors and bacterial FBD pathogens in the present study.

### 4.2. Interrelationships between FBD Pathogens and Influences of Climatic Factors

There is a complex interrelationship between natural environment factors and foodborne bacterial growth. Our study found that the incidence of FBD infections is strongly associated with climatic factors, such as temperature, relative humidity, and rainfall. In this study, the interrelationship network showed that salmonellosis, vibriosis, EHEC, EPEC, ETEC, and STA infections were influenced by insolation and cloudiness in addition to temperature, relative humidity, and rainfall (i.e., PC1 + PC2). Furthermore, the present study is the first to demonstrate that the triangular interrelationship of EPEC, ETEC, and STA with 5 climatic factors indicates an important interrelationship for these bacterial FBDs in the future as a result of climate change (Figure 2, red lines). Moreover, SAL–CAM and VBR–EHEC infections, respectively, were positively influenced by temperature, relative humidity, and rainfall (i.e., PC1) (Figure 2, purple lines). The positive interrelationship between FBD infectious cases and temperature, humidity, and rainfall in the present study is broadly consistent with previous studies on salmonellosis [1,5], campylobacteriosis [9], vibriosis [4], EHEC O157:H7 [8], and STA infection [30]. Relative humidity and rainfall also affect water and sanitation infrastructure and the number of pathogens and might affect the replication rate of certain foodborne pathogens [31].Notably, the positive relationship between temperature and Salmonella infections, observed in this study, using PCA and the PCCM technique, was similar to recent findings from Australia, Europe, North America, and Asia with similar trends [32]. Therefore, our results suggest that previous findings based on this assumption can be improved by assuming a non-stationary association and more accurately evaluating the possible non-linear association between climatic factors and FBD infectious transmission.

Meanwhile, CAM and CLP infections were negatively influenced by insolation and cloudiness (i.e., PC2) (Figure 2, blue lines). In other words, increased insolation and sunny weather mean that CAM and CLP infections could increase. Notably, Figure 2 shows that the risk of CAM is positively associated with temperature, relative humidity, and rainfall in our present study, which is broadly similar with previous studies [6,9,10]. For example, in Australia, an inverse relationship between weekly temperature and campylobacteriosis cases in Adelaide was shown, while a positive relationship was reported in Brisbane [33]. In addition, Lake (2017) [16] reported that the presented evidence that diseases caused by *Campylobacter* is associated with weather; FBD incidence is greater in the summer and during periods of warmer weather; hence, its incidence is also elevated. Schijven et al. [34] studied the relationship between *Campylobacter* and climate change using the quantitative microbial risk assessment (QMRA) approach. The results indicate that *Campylobacter* cases associated with poultry consumption are likely to increase under climate change, whereas risks associated with the drinking water pathway are likely to decrease due to increased inactivation in higher warmer temperatures. However, there is no direct interrelationship between CAM and CLP infections.

There are strong associations between Salmonella and the environment, especially temperature (Figure 2). However, even if SAL exhibited the most connections in this network, the relative abundance of cases did not appear to affect the interrelationships. In addition, Lake (2017) [16] reported that, in contrast to *Campylobacter*, there is a much clearer biological mechanism explaining why higher temperature leads to an elevated incidence of illness with Salmonella, i.e., at elevated ambient temperatures, Salmonella reproduction may be enhanced. Other studies, however, indicated that maximum and minimum temperatures, relative humidity, and rainfall were all positively correlated with the number of cases of Salmonella, with the lag values of the effects being between 2 weeks and 2 months [35,36,37]. These reported that rainfall, especially heavy rainfall events, may affect the frequency and level of contamination of drinking water and hence enteric infection. A strong association between drinking water quality, precipitation, and gastroenteritis was reported [38].

Among 12 foodborne pathogens, EIEC, BAC, LIS, and YER infections were not significantly influenced by any of the 5 climatic variables in South Korea. However, the lack of relationships between these FBD pathogens and climatic factors remains unclear. For example, LIS–STA had a slightly elevated Z-score, although this did not reach statistical significance (Table 2). Thus, these relationships may vary with respect to regional differences in the effects of climatic factors, possibly affecting the incidence of bacterial FBDs [19].

### 4.3. Limitations

To better understand these interrelationships, it would be interesting to investigate the time series—that is, by using Lotka–Volterra modeling as an example of a dynamic model of a microbial community [39,40]—considering the lag effects of meteorological variables on foodborne infection [7] as well as the spatial, regional, or worldwide distributions of these FBD infections with respect to multiple climatic factors. Moreover, the mechanisms and genetic effects involved in these interrelationships remain unclear. In particular, pathogenic *E. coli* exhibited different interrelationships with respect to serotype, necessitating further study. In addition, the incidences of some bacterial FBDs are largely dependent on food safety interventions and policies as well as human factors including behavior and consumption patterns [41]. These social, public health, and environmental relationships should also be considered alongside multiple climatic factors. Studies of vulnerability to FBDs and future climate change with respect to aging are also required.

Although we used PCA and the PCCM technique as a novel approach to identify possible interrelationships between FBD pathogens and climatic factors, these interrelationships must be confirmed by further studies with more robust data. Nevertheless, the results suggest that perturbed interrelationships or higher-order relationships among these climatic factors play an important role in the incidence of FBDs. Moreover, the interrelationships between multiple climatic factors and bacterial FBD incidence can be used to develop prediction models for future disease patterns with respect to climate change. However, determining the causality and directions of these relationships in the network presented herein requires further prospective and functional studies of these mechanisms to identify the interrelationships between FBD pathogens and additional climatic factors. We anticipate our method will serve as a foundation for more sophisticated prediction models and the identification of mechanisms of future patterns of FBD with respect to climate change.

## 5. Conclusions

In conclusion, this study could highlight the understanding of the interrelationship between climatic variables and FBD incidence by characterizing the association between multiple climate factors and the incidence of FBDs in South Korea. In addition, these results show climate forces as factors influencing enteric disease incidence, emphasizing the potential effect of future regional climate change on FBD risk. Notably, the combination of climatic factors including temperature, relative humidity, rainfall, insolation, and cloudiness emerged as potential forecast factors for salmonellosis, vibriosis, STA, and pathogenic *E. coli* infection. Thus, our results showed many pathways through which FBD may be affected by multiple climate factors. The statistical analysis used in this study could be useful for determining the effects of multiple climate factors on FBD patterns using QMRA. Moreover, the interrelationships between various climatic factors and the national incidences of bacterial FBD could contribute to the development of prediction models for future patterns of diseases based on climate change. However, multiple climatic factors may have direct or indirect effects on FBD, and consequently, there could be a huge degree of uncertainty as to the overall effect of multiple climate factors on FBD. Therefore, it is important to recognize that FBD usually occurs as a consequence of a combination of one or more specific climatic factors [42].

## Figures and Tables

**Table 1:** Partial correlation coefficients (*r_ij_*) of 12 bacterial FBD pathogens with respect to 3 climatic variable groups.

Variables		SAL	EPEC	ETEC	EIEC	EHEC	CAM	STA	CLP	VBR	LIS	BAC	YER
**PC1+PC2 (5 climatic factors)**	**SAL**	1.000											
	**EPEC**	−0.065	1.000										
	**ETEC**	0.078	0.330	1.000									
	**EIEC**	−0.016	0.056	−0.139	1.000								
	**EHEC**	0.116	0.055	0.147	0.129	1.000							
	**CAM**	−0.102	−0.126	−0.179	0.079	−0.135	1.000						
	**STA**	0.133	0.360	0.346	−0.029	−0.063	−0.099	1.000					
	**CLP**	−0.030	−0.052	−0.150	0.017	0.002	0.038	−0.140	1.000				
	**VBR**	0.285	0.017	0.018	0.210	0.084	−0.147	0.275	−0.289	1.000			
	**LIS**	−0.066	0.180	0.073	0.023	−0.001	−0.221	0.252	−0.005	0.170	1.000		
	**BAC**	−0.009	−0.021	0.050	0.035	0.037	0.077	−0.093	−0.024	0.141	−0.046	1.000	
	**YER**	−0.093	−0.040	0.038	−0.080	−0.070	−0.040	−0.069	−0.047	−0.125	−0.073	−0.141	1.000
**PC1**	**SAL**	1.000											
	**EPEC**	−0.120	1.000										
	**ETEC**	0.237	0.294	1.000									
	**EIEC**	0.044	0.048	−0.118	1.000								
	**EHEC**	0.390	0.009	0.227	0.145	1.000							
	**CAM**	0.473	−0.161	0.022	0.104	0.169	1.000						
	**STA**	0.136	0.350	0.352	−0.024	−0.029	−0.030	1.000					
	**CLP**	0.084	−0.064	−0.114	0.025	0.054	0.112	−0.130	1.000				
	**VBR**	0.542	−0.035	0.132	0.216	0.267	0.220	0.273	−0.187	1.000			
	**LIS**	−0.065	0.182	0.063	0.021	−0.014	−0.189	0.249	−0.009	0.132	1.000		
	**BAC**	−0.132	−0.005	0.010	0.024	−0.031	−0.045	−0.103	−0.044	0.043	−0.040	1.000	
	**YER**	−0.159	−0.027	0.006	−0.087	−0.116	−0.115	−0.078	−0.063	−0.172	−0.068	−0.118	1.000
**PC2**	**SAL**	1.000											
	**EPEC**	−0.023	1.000										
	**ETEC**	0.195	0.329	1.000									
	**EIEC**	−0.038	0.055	−0.143	1.000								
	**EHEC**	0.492	0.061	0.230	0.083	1.000							
	**CAM**	−0.394	−0.125	−0.245	0.087	−0.362	1.000						
	**STA**	0.202	0.360	0.366	−0.035	0.039	−0.157	1.000					
	**CLP**	−0.285	−0.059	−0.208	0.029	−0.210	0.196	−0.185	1.000				
	**VBR**	0.383	0.024	0.069	0.192	0.222	−0.248	0.303	−0.356	1.000			
	**LIS**	−0.029	0.180	0.075	0.022	0.012	−0.207	0.253	−0.013	0.170	1.000		
	**BAC**	0.051	−0.019	0.064	0.032	0.075	0.033	−0.080	−0.051	0.156	−0.044	1.000	
	**YER**	0.030	−0.036	0.062	−0.084	0.018	−0.093	−0.048	−0.089	−0.086	−0.069	−0.130	1.000

*r_ij_*: magnitude of a partial correlation coefficient between 2 variables: *i* and *j*. PC1: temperature, relative humidity, and rainfall, PC2: insolation and cloudiness. BAC: *B. cereus*; CAM: campylobacteriosis; CLP: *C. perfringens*; LIS: *Listeria* spp.; SAL: salmonellosis; EPEC, ETEC, EIEC, EHEC: pathogenic *E. coli* infection by enteropathogenic, enterotoxigenic, enteroinvasive, enterohemorrhagic *E. coli*, respectively; STA: *S. aureus*; VBR: *V. parahaemolyticus*; YER: *Y. enterocolitica* infection.

**Table 2:** Partial correlation coefficients matrix transformed to *Z*-terms (*Z_α_*_/2_) of 12 bacterial FBD pathogens with respect to 3 climatic variable groups.

Variables		SAL	EPEC	ETEC	EIEC	EHEC	CAM	STA	CLP	VBR	LIS	BAC	YER
**PC1+PC2 (5 climatic factors)**	**SAL**												
	**EPEC**	−0.56											
	**ETEC**	1.61	2.37 *										
	**EIEC**	0.00	0.38	−0.99									
	**EHEC**	3.58 **	0.29	1.80	0.85								
	**CAM**	0.32	−1.06	−0.91	0.72	−0.78							
	**STA**	1.29	2.76 **	2.85 **	−0.22	−0.01	−0.69						
	**CLP**	−0.57	−0.45	−1.20	0.20	−0.53	1.10	−1.17					
	**VBR**	4.10 **	−0.04	0.77	1.55	1.89	−0.14	2.24 *	−2.12 *				
	**LIS**	−0.41	1.37	0.52	0.16	0.00	−1.57	1.92	−0.08	1.12			
	**BAC**	−0.28	−0.09	0.27	0.21	0.20	0.03	−0.69	−0.34	0.72	−0.32		
	**YER**	−0.60	−0.24	0.25	−0.64	−0.37	−0.68	−0.47	−0.55	−0.98	−0.52	−0.95	
**PC1**	**SAL**												
	**EPEC**	−0.90											
	**ETEC**	1.81	2.27 *										
	**EIEC**	0.33	0.36	−0.89									
	**EHEC**	3.08 **	0.07	1.73	1.09								
	**CAM**	***3.84*** **	−1.22	0.17	0.78	1.28							
	**STA**	1.02	2.74 **	2.75 **	−0.18	−0.22	−0.22						
	**CLP**	0.63	−0.48	−0.86	0.19	0.40	0.84	−0.98					
	**VBR**	4.54 **	−0.26	0.99	1.64	***2.05*** *	1.67	2.10 *	−1.42				
	**LIS**	−0.49	1.38	0.47	0.16	−0.10	−1.43	1.91	−0.07	1.00			
	**BAC**	−1.00	−0.04	0.07	0.18	−0.23	−0.33	−0.77	−0.33	0.32	−0.30		
	**YER**	−1.20	−0.20	0.04	−0.66	−0.87	−0.86	−0.58	−0.47	−1.30	−0.51	−0.88	
**PC2**	**SAL**												
	**EPEC**	−0.17											
	**ETEC**	1.48	2.55 *										
	**EIEC**	−0.29	0.41	−1.08									
	**EHEC**	4.03 **	0.46	1.76	0.62								
	**CAM**	−3.12 **	−0.94	−1.87	0.65	−2.83 **							
	**STA**	1.54	2.82 **	2.87 **	−0.26	0.29	−1.18						
	**CLP**	−2.19 *	−0.44	−1.58	0.22	−1.60	1.49	−1.40					
	**VBR**	3.02 **	0.18	0.52	1.46	1.69	−1.89	2.34 *	−2.79 **				
	**LIS**	−0.22	1.36	0.56	0.17	0.09	−1.57	1.93	−0.09	1.28			
	**BAC**	0.38	−0.14	0.48	0.24	0.57	0.25	−0.60	−0.38	1.18	−0.33		
	**YER**	0.23	−0.27	0.46	−0.63	0.13	−0.70	−0.36	−0.67	−0.65	−0.52	−0.98	

PC1: temperature, relative humidity, and rainfall; PC2: insolation and cloudiness. BAC: *B. cereus*; CAM: campylobacteriosis; CLP: *C. perfringens*; LIS: *Listeria* spp.; SAL: salmonellosis; EPEC, ETEC, EIEC, EHEC: pathogenic *E. coli* infection by enteropathogenic, enterotoxigenic, enteroinvasive, enterohemorrhagic *E. coli*, respectively; STA: *S. aureus*; VBR: *V. parahaemolyticus*; YER: *Y. enterocolitica* infection. * *p* < 0.05; ** *p* < 0.01. Bold italics: coefficients altered by 3 conditional climatic variable groups (PC1 + PC2 to PC1 to PC2). Colored cells indicate significant interrelationships. Orange: 7 interrelationships under PC1 + PC2; purple: 2 added (SAL-CAM and EHEC-VBR) interrelationships under PC1; blue: 4 (3 added (SAL-CLP, EHEC-CAM, and CLP-VBR) and 1 changed (SAL-CAM)) interrelationships under PC2.
